# Expression of two parental imprinted miRNAs improves the risk stratification of neuroblastoma patients

**DOI:** 10.1002/cam4.264

**Published:** 2014-06-13

**Authors:** Charles-Henry Gattolliat, Gwénaël Le Teuff, Valérie Combaret, Eugénie Mussard, Dominique Valteau-Couanet, Pierre Busson, Jean Bénard, Sétha Douc-Rasy

**Affiliations:** 1 CNRS UMR 8126, Université Paris-SudGustave Roussy, 114 rue Edouard Vaillant, 94805, Villejuif, France; 2 INSERM UMR 1078, Etablissement Français du Sang, Centre Hospitalier Régional Universitaire de Brest, SFR ScInBioS, Université de Bretagne Occidentale, Faculté de Médecine22 avenue Camille Desmoulins, 29200, Brest, France; 3 Department of Biostatistics and Epidemiology, Gustave Roussy114 rue Edouard Vaillant, 94800, Villejuif, France; 4 Laboratoire de Recherche Translationnelle, Centre Léon Bérard69373, Lyon Cedex 08, France; 5 Paediatric Department, Gustave Roussy114 rue Edouard Vaillant, 94805, Villejuif, France; 6 Department of Bio-Pathology, Gustave Roussy114 rue Edouard Vaillant, 94805, Villejuif, France

**Keywords:** miRNA clusters, neuroblastoma, parental imprinting, prognostic model, risk score, survival outcomes

## Abstract

Age at diagnosis, stage, and *MYCN* amplification are the cornerstones of the risk-stratification score of neuroblastoma that enables defining patients at low- and high risk. Refinement of this stratification is needed to optimize standard treatment and to plan future clinical trials. We investigated whether two parental imprinted miRNAs (miR-487b and miR-516a-5p) may lead to a risk score with a better discrimination. Expression levels of maternal miR-487b and paternal miR-516a-5p were determined using quantitative RT-PCR both for 231 neuroblastoma tumors (derivation set) and 101 independent neuroblastoma tumors (validation set). Survival outcomes were overall survival (OS) and disease-free survival (DFS). Multivariable Cox models were developed from derivation set and their performance evaluated using Akaike's information criterion (AIC) (goodness-of-fit) and time-dependent area under curves (discrimination). The selected model was validated using internal and external validation. The prognostic model including current prognostic factors plus miR-487b, miR-516a-5p, and interaction between two miRNAs was selected. Performance of this model was better in terms of both predictive ability (smallest AIC) and discrimination power (AUC close to 0.70). This model identifies three risk groups: high (3), intermediate (2), and low (1). Hazard ratios (HR) across risk groups were HR_2/1_ = 6.3 (2.7–14.6), HR_3/1_ = 14.8 (7.2–30.2) for OS and HR_2/1_ = 2.8 (1.5–5.4), HR_3/1_ = 7.2 (3.9–13.4) for DFS. The rank between these three risk groups was maintained and validated when performing internal and external validation. Expression of maternal miR-487b and paternal miR-516a-5p improves the risk stratification. This better discrimination at diagnosis is of clinical utility both for current and future treatments of neuroblastoma patients.

## Introduction

Neuroblastoma, a malignant solid tumor of neural crest origin, accounts for 10% of all childhood cancers and exhibits a high clinical heterogeneity 1. The combination of age at diagnosis, tumor stage, and *MYCN* amplification status constitutes the score of the standard algorithm at diagnosis that enables to stratify patients into low- and high-risk groups, for both overall survival (OS) and disease-free survival (DFS) 2,3. The low-risk group (up to 90% survival at 5 years) consists of non-*MYCN*-amplified tumors that are either localized (stages 1, 2, and 3) or metastatic in children <18 months old (stages 4 and 4S). The high-risk group (around 35% survival at 5 years) comprises all cases *MYCN*-amplified neuroblastoma regardless of stage and age, plus non-*MYCN*-amplified stage 4 tumors in children ≥18 months old. Additional prognostic markers of relapse for low-risk patients and of survival for high-risk patients are required to optimize the management of neuroblastoma and to improve outcome for the patients 1,4. Several attempts had been made to sort out molecular tumor markers of prognostic significance based on nucleic acids machinery (DNA, RNA) or biochemical traits 5,6. So far the pan-genomic algorithm (gain or loss of whole chromosomes and intrachromosomal alterations) has been shown to improve the standard risk stratification 7,8. On the other hand, several reports have identified gene expression patterns to predict the outcome of neuroblastoma patients 2,9. Recently, Valentijn and colleagues defined a 157-genes signature that predicts clinical outcome of neuroblastoma irrespective of *MYCN* amplification 10.

MicroRNAs, due to their current exhaustive identification and chemical stability, represent another option to identify putative prognostic markers. These small RNAs interfere with diverse biological functions through the post-transcriptional regulation of gene expression by acting on the stability or translational rate of mRNA 11. The involvement of miRNAs in tumor initiation and progression is well established and offers new perspectives in the therapeutic management of neuroblastoma patients 12,13. Some recent works have been published in neuroblastoma patients. Each study proposed different miRNA signatures associated to neuroblastoma prognosis but not compared to the current classification systems 14–17. De Preter and colleagues have proposed a 25-miRNA signature able to identify a cohort of high-risk neuroblastoma patients at greater risk of poor outcome 18.

We previously showed the prognostic role of miR-487b beyond the current risk factors in neuroblastoma tumors 19. This miRNA belongs to the largest imprinted miRNA cluster (C14MC) at the 14q32.31 locus. Besides neuroblastoma tumors, alterations of expression of C14MC are frequently found in many other human cancers and diseases 20,21. In human, the second large imprinted miRNA cluster, called chromosome 19 microRNA cluster (C19MC), locates at the 19q13.4 locus 22,23. Several studies showed overexpression of C19MC miRNAs as associated with poor prognosis of many tumors 24,25.

In this study, we extended the work of Gattolliat and coworkers 19 in evaluating the prognostic of the expression of only two miRNAs, each representing the most significant prognostic marker of C14MC and C19MC, the two imprinted clusters. To this purpose, we evaluated the added prognostic information from miR-487b and miR-516a-5p to the current prognostic factors (age, stage, *MYCN*) on the survival outcomes.

## Materials and Methods

### Tumor's collection, cryosections, and total RNA extraction

Tumors samples of a retrospective cohort, partially previously published by Gattolliat and colleagues 19, were gathered, between 1987 and 2009, at the *Gustave Roussy*, Villejuif, France (*n* = 223) and at the *Centre Léon Bérard*, Lyon (*n* = 8). This cohort was referred to as derivation set. An independent cohort of 101 patients from *Centre Léon Bérard* followed between 1988 and 2010 was constructed for the external validation (validation set). All tumors samples were collected with the approval of the appropriate ethic committees, according to the national law on the protection of people taking part in biomedical research. Patients were staged according to the International Neuroblastoma Staging System 26. Storage of primary tumor tissues, cryosections, and total RNA isolation were previously described 19. The first and last cryosections were used to select tumor tissues with a malignant tumor cell content of ≥60% 1,27.

### miRNA quantitative RT-PCR

The quantitative real-time polymerase chain reaction (qRT-PCR) and statistical analyses were done as previously described 19. Briefly, qRT-PCR was performed according to the MIQE guidelines 28 and the quantification method was done according to Livak and Schmittgen 29. RNU-44 was used as normalizer and IGR-N-835 cell line as calibrator 30. The statistical analyses compared the miRNA expression level between low- and high risk using the two-tailed Student's *t*-test. These analyses included a reanalysis of miR-487b previously identified 19 as well as an analysis of miR-516a-5p, the most significant miRNA marker from the C19MC cluster.

### Statistical analysis

The median follow-up was calculated using the Schemper's method 31. OS and DFS were defined as the time from the diagnosis to death and to the first occurrence of relapse or death, respectively. Survival curves were estimated using the Kaplan–Meier method and were compared with the log-rank test. Five multivariable Cox proportional hazard models were developed to assess individually (or not) the added prognostic information of two markers: one from C14MC and the second from C19MC. Model 1 included the three current prognostic factors and was later referred to as the reference model. Models 2 and 3 were built in adding one miRNA to the reference model. Model 4 was the reference model plus the two miRNAs. This model was extended including an interaction term between the two markers (model 5). All models were compared using the likelihood test ratio and Akaike's information criterion (AIC). The discriminant performance of models was measured using the time-dependent area under the curve (AUC) using the *SurvivalROC* R package 32. This criterion allows evaluating whether a model may correctly classify a patient with an event and a patient without event at a time point; AUC close to 1 indicating a better classification. The performance of the final model was also evaluated in deriving risk groups from the prognostic index (PI) (sum of weighted regression coefficients estimated from derivation set). The discrimination of the observed survival curves of risk groups (Kaplan–Meier) and the comparison with final model-based predicted mean survival curves were studied. The final model was evaluated through internal and external validation. The first validation used a leave-one-out cross-validation that allowed to validate the development process of the prognostic model 33. The external validation enabled evaluating the generalization of the results. Whatever the type of validation, we estimated the PI in relying on (1) reuse of derivation set for internal validation (PICV) and (2) the validation set for external validation (PIV). In this aim, using the regression coefficients estimated (1) from the estimation set for the internal validation and (2) from the derivation set for the external validation, we derived three risk groups in categorizing the continuous PI estimated previously using cut-points based on the distribution of (1) PICV for the internal validation and (2) PI across the individual in the derivation set. The choice of three groups was arbitrary, but when two were chosen (low- and high-risk groups), this was not sufficient to the routine clinical practice; and, choosing four, may be too large regarding the number of events 33. We estimated the observed survival curves (Kaplan–Meier) into the three risk groups and evaluated whether they were well separated and reported the hazard ratios across these risk groups. The comparison with the predicted survival curves allowed evaluating the accuracy prediction of the model (calibration). Less importance was attached to this point since our aim was to define a risk score with good ability to separate patients with different prognoses. An update of the final prognostic model was performed on the pool of the derivation and validation set with adjustment by dataset, thus providing more accurate estimate of regression coefficients. For all analyses, we categorized continuous miRNA expression levels as measured by qRT-PCR into two classes (<1, ≥1), using as cutoff the expression level of IGR-N-835 cell line taken as one 19. This cutoff was found to be very close to those computed for the miRNA expression levels that best discriminate patients for survival (overall and DFS) of patients (data not shown). All tests were two-sided with a nominal significance level of 5% and statistical analyses were carried out with SAS® version 9.3 software (SAS Institute, Inc., Cary, NC).

## Results

### Description of the derivation and validation sets

**Figure 1:**
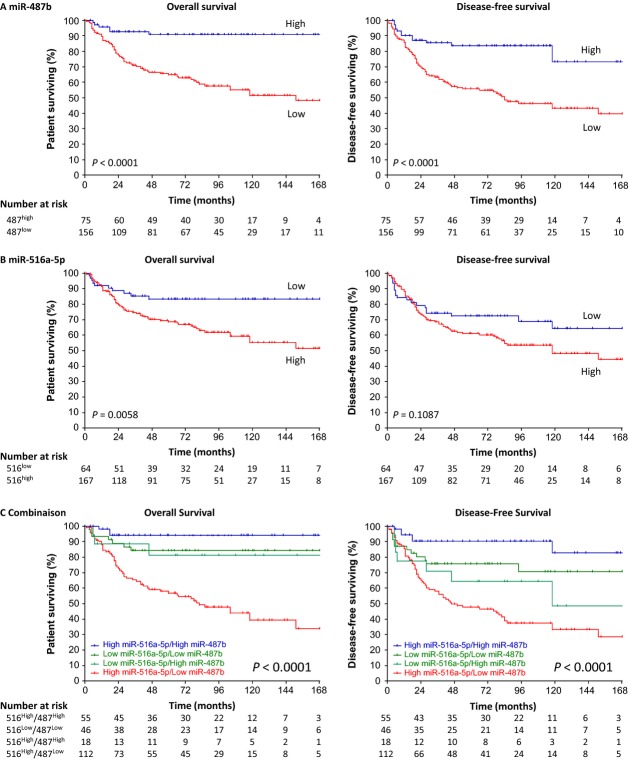
(A) Kaplan–Meier curves for overall survival (left) and disease-free survival (right) of the derivation set (*n* = 231) for miR-487b. miRNA expression levels were converted into discrete variables by discriminating the samples into two classes (high- and low expression), under or over the cutoff defined as the expression level of IGR-N-835 cell line taken as one. (B) Kaplan–Meier curves for overall survival (left) and disease-free survival (right) of the derivation set (*n* = 231) for miR-516a-5p. miRNA expression levels were converted into discrete variables by discriminating the samples into two classes (high- and low expression), under or over the cutoff defined as the expression level of IGR-N-835 cell line taken as one. (C) Kaplan–Meier curves for overall survival and disease-free survival of the derivation set (*n* = 231) for the four combinations of miRNA expression: “miR-516a-5p^high^/miR-487b^high^”, “miR-516a-5p^high^/miR-487b^low^”, “miR-516a-5p^low^/miR-487b^high^”, and “miR-516a-5p^low^/miR-487b^low^”.

**Figure 2:**
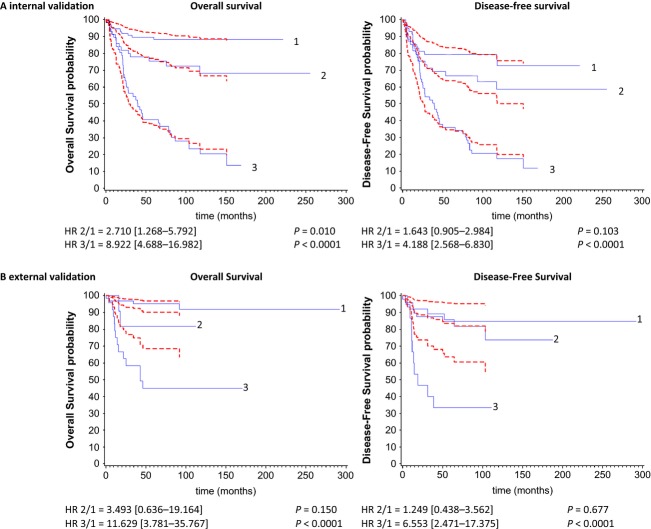
Comparing observed (Kaplan–Meier) survival curves for risk groups (solid lines) and model-based predicted mean survival curves (dashed lines) (A) for the leave-one-out cross-validation (*n* = 231) and (B) for the validation set (*n* = 101). Below each figure, the hazard ratio (HR) and 95% confidence interval (95% CI) of prognostic risk sets using group 1 as baseline were reported. The different risk groups were defined as follows: 1 = “miR-516a-5p^high^/miR-487b^high^”, 2 = “miR-516a-5p^low^/miR-487b^high and low^”, 3 = “miR-516a-5p^high^/miR-487b^low^”.

Main characteristics of the two tumor sets used in our study are reported in Table1, and individual patient data are presented in Table S1. The derivation set of 231 neuroblastoma patients was followed until March 2012. The median follow-up was 7.7 years (95% confidence interval [CI] = [6.8–8.7]). According to the standard algorithm of disease outcome based on age at diagnosis, stage, and *MYCN* amplification, 65% (*n* = 151) patients were classified at low risk and 35% (*n* = 80) at high risk in the derivation set. The observed number of deaths was 69 (30%) and the number of events (death or relapse) was 90 (39%). In the external validation set (*n* = 101), the median follow-up of 6.7 years (5.4–8.0) was of the same order of magnitude of the derivation set. If patients were younger (*P *=* *0.05) with higher number of *MYCN* amplification (*P *=* *0.04) in the validation set, no significant difference into the standard risk stratification between the two datasets was observed (*P *=* *0.38). There is no significant difference between derivation and validation set in terms of OS (*P *=* *0.06) contrary to DFS (*P *=* *0.04) (Fig. S1 reporting the log-rank test). These last two comparisons were performed after controlling for stage, age, and *MYCN* amplification but considering the analysis of retrospective data some important omitted confounders; for example, the difference of treatments in the institutions where these archival cohorts were obtained, could explain the observed difference in term of DFS. Figure1A shows the updated survival curves reported in Gattolliat and coworkers with a significant log-rank test (*P *<* *0.0001 for both OS and DFS). Indeed, within the cluster C14MC, miR-487b was the miRNA whose the expression was the most associated with both OS (*P *=* *0.0018) and DFS (*P *=* *0.0003) (Figs.2 and 3, Table S2). The update of prognostic effect of miR-487b on OS and DFS reported in Gattolliat and colleagues is presented in column “model 2” of Table2.

**Table 1:** Main characteristics of patients in derivation and validation sets.

	Derivation set (*n* = 231)	Validation set (*n* = 101)	Total (*n* = 332)
Inclusion period	1987–2009	1988–2010	1987–2010
Stage^1^			
1	34 (15%)	29 (29%)	63 (19%)
2	47 (20%)	15 (15%)	62 (19%)
3	50 (22%)	20 (20%)	70 (21%)
4	81 (35%)	28 (29%)	109 (33%)
4S	19 (8%)	7 (7%)	26 (8%)
Age at diagnosis			
<18 months	110 (48%)	60 (59%)	170 (51%)
≥18 months	121 (52%)	41 (41%)	162 (49%)
*MYCN* status			
Nonamplified	201 (87%)	79 (78%)	280 (84%)
Amplified	30 (13%)	22 (22%)	52 (16%)
Standard risk stratification^2^			
Low	151 (65%)	71 (70%)	222 (67%)
High	80 (35%)	30 (30%)	110 (23%)
Overall survival			
Alive	162 (70%)	82 (81%)	244 (74%)
Dead	69 (30%)	19 (19%)	88 (26%)
Disease-free survival			
No event	141 (61%)	77 (76%)	218 (66%)
Event	90 (39%)	24 (24%)	114 (34%)

1 In the validation set, two patients have a missing stage.

2 Standard risk stratification is based on age at diagnostic, stage, and *MYCN* status.

**Table 2:** Multivariable Cox regression analyses and performance measurements of five prognostic models in derivation set (*n* = 231).

Characteristics	Model 1	Model 2	Model 3	Model 4	Model 5
*Overall survival*					
Age at diagnosis					
<18 months (reference)	1.000	1.000	1.000	1.000	1.000
≥18 months	2.037 (1.077–3.850)	1.929 (1.020–3.648)	1.917 (1.009–3.644)	1.817 (0.954–3.462)	1.957 (1.007–3.802)
*INSS* stage					
1, 2, 3, 4S (reference)	1.000	1.000	1.000	1.000	1.000
4	4.142 (2.231–7.688)	3.550 (1.903–6.624)	4.026 (2.156–7.519)	3.304 (1.747–6.247)	3.027 (1.588–5.769)
*MYCN* status					
Nonamplified (reference)	1.000	1.000	1.000	1.000	1.000
Amplified	1.813 (1.045–3.145)	1.552 (0.890–2.707)	1.837 (1.056–3.196)	1.570 (0.898–2.745)	1.605 (0.915–2.817)
MiR-487b					
Low (reference)		1.000		1.000	1.000
High miR-516a-5p		0.332 (0.140–0.789)		0.313 (0.131–0.746)	1.831 (0.461–7.267)
Low (reference)			1.000	1.000	1.000
High			1.851 (0.940–3.642)	1.983 (1.004–3.918)	2.773 (1.251–6.147)
Interaction term^1^					1.000
					0.091 (0.015–0.553)
Performance measurements					
−2 log(likelihood) (AIC)	631.527 (637.527)	623.498 (631.498)	627.891 (635.891)	619.002 (629.002)	612.851 (624.851)
*Disease-free survival*					
Age at diagnosis					
<18 months (reference)	1.000	1.000	1.000	1.000	1.000
≥18 months	1.547 (0.931–2.573)	1.482 (0.891–2.466)	1.501 (0.898–2.509)	1.424 (0.851–2.383)	1.556 (0.910–2.660)
*INSS* stage					
1, 2, 3, 4S (reference)	1.000	1.000	1.000	1.000	1.000
4	2.686 (1.631–4.422)	2.376 (1.435–3.933)	2.676 (1.622–4.413)	2.329 (1.400–3.875)	2.093 (1.246–3.516)
*MYCN* status					
Nonamplified (reference)	1.000	1.000	1.000	1.000	1.000
Amplified	1.427 (0.841–2.422)	1.252 (0.734–2.135)	1.430 (0.842–2.427)	1.249 (0.732–2.132)	1.283 (0.749–2.199)
miR-487b					
Low (reference)		1.000		1.000	1.000
High		0.455 (0.247–0.839)		0.440 (0.238–0.815)	2.100 (0.809–5.447)
miR-516a-5p					
Low (reference)			1.000	1.000	1.000
High			1.239 (0.741–2.071)	1.334 (0.795–2.239)	2.046 (1.095–3.822)
Interaction term^1^					1.000
					0.104 (0.029–0.371)
Performance measurements					
−2 log(likelihood) (AIC)	861.985 (867.985)	854.645 (862.645)	861.293 (869.293)	853.392 (863.392)	841.590 (853.590)

AIC, Akaike's information criterion.

1 See Table3 for a reformulation of interaction term in Model 5; Model 1: age + stage + mycn; Model 2: model 1 + miR-487b; Model 3: Model 1 + miR-516a-5p; Model 4: Model 1 + miR-487b + miR-516a-5p; Model 5: Model 1 + miR-487b + miR-516a-5p + miR-487b × miR-516a-5p.

### Strong expression of miR-516a-5p from the C19MC is associated with high-risk neuroblastoma

Previous miRNA microarray profiling of a screening cohort of 13 tumors (eight high-risk and five low-risk) revealed that miRNAs from C19MC were globally overexpressed in high-risk neuroblastomas 19, in agreement with observations from other high-grade tumors of various cancers 25. Among the microRNA of the C19MC cluster, miR-516a-5p was the only microRNA whose expression was significantly associated with survival. These results, confirmed by qRT-PCR in the derivation set (*n* = 231), showed that miR-516a-5p expression levels were higher in high-risk tumors (*P *=* *0.0091, Fig. S4). Kaplan–Meier analyses showed that patients with high expression levels of miR-516a-5p showed a shorter OS (*P *=* *0.0058) but not a shorter DFS (*P *=* *0.1087), compared to patients with low expression levels of this marker (Fig.1B). In terms of risk of outcomes for miR-516a-5p, OS was marginally associated (HR = 1.851, 95% CI = [0.940–3.642], *P *=* *0.0748), but DFS was not (HR = 1.239, 95% CI = [0.741–2.071], *P *=* *0.4144) when controlling for the current prognostic factors, that is, age, stage, and *MYCN* (column “model 3” in Table2). These hazard ratios were in the opposite direction compared to those of miR-487b. In addition, these two miRNAs were not significantly correlated: the percentage of high expression of miR-516a-5p was 70.9% and 75.3% in low and high expression of miR-487b, respectively (*P *=* *0.482).

### miR-487b and miR-516a-5p add prognostic information to the current prognostic factors

Figure1C showed a global significant difference (*P *<* *0.0001 for OS and DFS) between the four survival curves defined by the combination of miR-487b and miR-516a-5p. In particular, we observed that the difference between miR-487b-based survival curves was not significant in patients with low miR-516a-5p expression (*P *=* *0.8282 and *P *=* *0.3652 for OS and DFS, respectively), while it was significant in patients with high miR-516a-5p expression (*P *<* *0.0001 for OS and DFS). This suggests a modifier effect of miR-516a-5p on the prognostic value of miR-487b. The gain obtained with these two miRNAs may also be observed in the two subgroups defined by the standard risk stratification (low- and high risk). Within each risk group, expression of these two miRNAs allowed yet to identify patients with different prognoses (Fig. S5). The analysis of multivariable Cox models (reference model, on one hand, models including miR-487b and miR-516a-5p separately and together, on the other hand) measuring the adjusted associations of each prognostic factor with the hazard of survival outcomes is reported in Table2. Each model corresponds to a separate column in the table. The models including both miR-487b and miR-516a-5p for OS (column “model 4”) and including only miR-487b for DFS (column “model 2”) fit the data considerably better than the reference model: likelihood ratio test *P *=* *0.002 with lowest AIC = 629.002 for OS and *P *=* *0.007 with lowest AIC = 862.645 for DFS compared to the reference model. In these two models, high expression of miR-487b is associated with a 69% decrease in the hazard of death (HR = 0.31, 95% CI = [0.13–0.75]) and 54% decrease in the hazard of disease-related event (HR = 0.46, 95% CI = [0.25–0.84]). High expression of miR-516a-5p is associated with a 98% increase in the hazard of death (HR = 1.98, 95% CI = [1.00–3.92]). In “model 5,” a significant interaction term (*P *=* *0.013 and 0.0005 for OS and DFS, respectively) leads to a better predictive ability representing by the lowest AIC (AIC = 624.851 and 853.590 for OS and DFS, respectively) compared to all other models. In Table3 which reformulates model 5 reported in Table2, miR-516a-5p was a significant predictor of OS (HR = 2.77, 95% CI = [1.25–6.15], *P *=* *0.0120) and DFS (HR = 2.05, 95% CI = [1.09–3.82], *P *=* *0.0248), while miR-487b was significantly associated both with OS (HR = 0.17, 95% CI = [0.05–0.55], *P *=* *0.0031) and DFS (HR = 0.22, 95% CI = [0.09–0.52], *P *=* *0.0005) for high expression of miR-516a-5p. In addition, we evaluated the discriminant ability of the two models retained above, that is, including both miR-516a-5p and miR-487b (± interaction term) in comparing their time-dependent AUCs with that of the reference model. Table4 represents the time-dependent AUC within the first 5 years (see Fig. S6 for the time-dependent AUCs from five models until 10 years). The addition of the two miRNAs (with or without interaction) gave better risk discrimination than did the reference model, as shown by larger AUC leading to a relative increase which ranges from 8% to 62%. This suggests the potential of these prognostic models to identify groups of patients with different risks of event. Although model 5 had slightly lower discriminant ability than model 4, it was considered as the final model given it showed the lowest AIC criterion (overall performance). From it, three risk groups with significant different prognoses were identified: worse, intermediate, and good prognosis in the groups 3, 2, and 1, respectively (Fig. S7).

**Table 3:** Multivariable Cox regression model with two miRNAs plus interaction term controlling for current prognostic factors in derivation and combined sets.

Characteristics		Derivation set (*n* = 231)	Combined^1^ (*n* = 332)
*Overall survival*			
Age at diagnosis	<18 months (reference)	1.000	1.000
	≥18 months	1.957 (1.007–3.802)	1.760 (1.007–3.075)
*INSS* stage	1, 2, 3, 4S (reference)	1.000	1.000
	4	3.027 (1.588–5.769)	3.678 (2.073–6.523)
*MYCN* status	Nonamplified (reference)	1.000	1.000
	Amplified	1.605 (0.915–2.817)	1.583 (0.965–2.596)
miR-487b	Low (reference)	1.000	1.000
in miR-516a-5p^low^	High	1.831 (0.461–7.267)	1.327 (0.398–4.419)
miR-487b	Low (reference)	1.000	1.000
in miR-516a-5p^high^	High	0.167 (0.051–0.547)	0.266 (0.117–0.606)
miR-516a-5p	Low (reference)	1.000	1.000
	High	2.773 (1.251–6.147)	2.809 (1.391–5.672)
*Disease-free survival*			
Age at diagnosis	<18 months (reference)	1.000	1.000
	≥18 months	1.556 (0.910–2.660)	1.462 (0.925–2.310)
*INSS* stage	1, 2, 3, 4S (reference)	1.000	1.000
	4	2.093 (1.246–3.516)	2.658 (1.667–4.238)
*MYCN* status	Nonamplified (reference)	1.000	1.000
	Amplified	1.283 (0.749–2.199)	1.267 (0.799–2.009)
miR-487b	Low (reference)	1.000	1.000
in miR-516a-5p^low^	High	2.100 (0.809–5.447)	1.242 (0.518–2.978)
miR-487b	Low (reference)	1.000	1.000
in miR-516a-5p^high^	High	0.219 (0.093–0.517)	0.317 (0.166–0.604)
miR-516a-5p	Low (reference)	1.000	1.000
	High	2.046 (1.095–3.822)	1.905 (1.104–3.287)

1 In the validation set, two patients have a missing stage.

**Table 4:** Area under the curve (relative variation in %) at different time points^1^ from the three prognostic models (reference model, model including miR-487b and miR-516a-5p and model including both these two markers and interaction term) in derivation set (*n* = 231).

Time (months)	12	24	36	48	60
Overall survival					
Model 1	0.45	0.47	0.49	0.56	0.56
Model 4	0.64 (42%)	0.75 (62%)	0.76 (54%)	0.79 (43%)	0.79 (42%)
Model 5	0.59 (31%)	0.70 (51%)	0.71 (43%)	0.75 (36%)	0.75 (35%)
Disease-free survival					
Model 1	0.46	0.46	0.49	0.56	0.57
Model 4	0.52 (13%)	0.64 (40%)	0.67 (35%)	0.71 (27%)	0.71 (26%)
Model 5	0.49 (8%)	0.62 (35%)	0.67 (34%)	0.73 (29%)	0.74 (29%)

Each model was controlling for the current prognostic factors. Model 1: age + stage + mycn; Model 4: Model 1 + miR-487b + miR-516a-5p; Model 5: Model 1 + miR-487b + miR-516a-5p + miR-487b × miR-516a-5p.

1 For each time point, the status of patients was defined as 1 if *T* ≤ *t* and 0, T > *t* and compute the AUC at this time.

### Internal and external validations provide a prognostic model including two miRNAs plus interaction term

The order of risk groups derived from the derivation set was maintained in the internal and external validation. The categorization of PICV (Prognostic Index for Internal Validation) according to its median and third quartile indicated good separation of three groups (Fig.2A). This discrimination is characterized by the HRs across risk groups (HR_2/1_ = 2.710, 95% CI = [1.268–5.792] and HR_3/1_ = 8.922, 95% CI = [4.688–16.982] for OS; and HR_2/1_ = 1.643, 95% CI = [0.905–2.984] and HR_3/1_ = 4.188, 95% CI = [2.568–6.830] for DFS). This model also fitted well with similarity between the observed (Kaplan–Meier) and predicted survival curves (calibration attribute) (Fig.2A). We confirmed the three prognostic groups in the validation set (Fig.2B) with a similar order between them even if the discrimination was less marked between the intermediate (group 2) and good (group 1) prognostic groups and more particularly for DFS (see Table S3 for PI equation and the cutoff defining the risk groups). It may be possibly explained by a better DFS in patients of the validation set making difficult to separate patients into risk sets of different prognoses. Another reason is the limited number of events in the validation set (Table1). The HRs of prognostic risk sets were HR_2/1_ = 3.493, 95% CI = (0.636–19.164) and HR_3/1_ = 11.629, 95% CI = (3.781–35.767) for OS; and HR_2/1_ = 1.249, 95% CI = (0.438–3.562) and HR_3/1_ = 6.553, 95% CI = (2.471–17.375) for DFS. In term of calibration, the inaccuracy prediction between observed and predicted survival curves may result by substantial different baseline survival function in the two populations (derivation and validation sets). This last point is, however, less important regarding the aim focusing on a risk score able to well-discriminate neuroblastoma patients. We also provided an update of regression coefficients of model 5 (adjusted by dataset) in carrying out a pooling of the two datasets. Column “Combined” of Table3 shows similar regression coefficients.

## Discussion

We have identified two parental imprinted miRNAs (miR-487b and miR-516a-5p) able to improve the current risk stratification of neuroblastoma based on age, stage, and *MYCN* amplification. The addition of these two markers with these three current prognostic factors improves the predictive ability and allows identifying new groups of patients with different prognoses both in terms of DFS and OS. On a basic point of view, these two markers belong to the two imprinted clusters, one maternally imprinted, C14MC, and the other paternally imprinted, C19MC, both clusters located at fragile sites of the human genome. Given the monoallelic expression of these clusters in normal cells, one can assume that any variation in expression of microRNAs of these loci (increase or decrease) may be instrumental for oncogenesis. As a matter of fact, high-risk neuroblastomas show differential underexpression of miR-487b sharply contrasting to the overexpression of miR-516a-5p. Very recently, the role of miR-487b as tumor suppressor gene was assigned: indeed, a loss of miR-487b expression was shown during the carcinogenesis of airway epithelial cells 34. But so far regarding function of miR-516a-5p, nothing at all is known. Therefore, it would be attractive to consider miR-516a-5p as an actor playing an oncogenic role.

However, in considering the combinations of miR-487b/miR-516a-5p expressions in various risk subtypes that our study delineates herein, it seems to be more appropriate to speculate for miR-516a-5p a role of a fine-tuning regulator of neuroblastoma oncogenesis. Besides neuroblastoma tumors, alterations of expression of either C14MC 20,21 or C19MC 25 are frequently found in many other human cancers. In relationship with neural crest origin and more particularly in neuroectodermal brain tumors of children, overexpression of a set of microRNAs from the C19MC (related to an amplification of the locus) hallmarked aggressive primitive tumors 35. As demonstrated over the last 5 years, miRNAs have been identified as interesting prognostic markers in neuroblastoma 14–17. One important advantage of miRNAs is their high stability as compared to mRNA that limits the clinical routine application of gene expression 36,37. De Preter and colleagues proposed a global signature based on 25 miRNAs 18. These authors evaluated the discriminant ability of this signature and obtained AUC values of 0.74 for OS and 0.72 for DFS with an endpoint to 3 years. We obtained similar results with AUC at 3 years estimated to 0.71 and 0.67 for OS and DFS, respectively, using a parsimonious model into account six parameters to estimate (age, stage, *MYCN*, miR-487b, miR-516a-5p, and interaction between the two markers). Noteworthy, miR-487b and miR-516a-5p do not belong to the signature of De Preter and coworkers. However, no functional studies warrant identity of target genes, so far. Whether or not our two miRNAs and this signature would regulate similar key genes involved in pathways related to neuroblastoma oncogenesis remain to be determined. The discriminant ability of our model, estimated from the derivation set, was also confirmed by an external validation set of 101 patients, albeit at a lesser extent between intermediate- and low-risk groups. This may be likely explained by a relative homogeneity of these patients in terms of prognosis. This evidence allows foreseeing the generalization of the model.

In a daily clinical practice, the quantification of two miRNAs expression is realistic and feasible in an easy and rapid way using quantitative RT-PCR; not to mention this analysis can be done with a low number of cells such as that resulting from fine needle aspirations. Our results were obtained from tumor tissues with a malignant tumor cell content of ≥60%. In this respect, similarly to global genomic approaches (array-comparative genomic hybridization (CGH) and genes expression panel), the routine application of qRT-PCR analysis in clinical practice might be considerably limited by stromal cells contamination. So the RT-PCR analysis must be completed with single-cell methods, such as fluorescence in situ hybridization, to evaluate the results concordance and the clinical implications. With regard to stratification based on pan-genomic alterations, up to now, the published studies of reference considering on one hand seven factors (segmental alterations, stage, age, *MYCN* amplification, 1p deletion, 11q deletion, and 1q gain) 7, on the other hand more than 12 genomic abnormalities 8 did not refer their discriminant ability onto AUC value. Thus, it would urge, in studying the same large tumor cohort, to estimate AUC at different times from genomic DNA analysis and compare them with those obtained with our prognosis model. Whether the combination of miRNAs and pan-genomic alterations constitute an algorithm that could improve the risk stratification of neuroblastoma remains to be investigated.

Further studies on larger external cohorts are needed to definitively assess the role of these two markers in neuroblastoma and to investigate whether the association between the two miRNA from the C14MC and C19MC in the tumor progression of neuroblastoma may be extended to other specific miRNA from these clusters in various human cancers. Also and importantly, future studies will ascertain the impact of the combined miR-487b/miR-516a-5p expression on the genomic stratification neuroblastoma through CGH-array 7,38. Such findings would open to a better treatment (therapeutic escalation or des-escalation).

In conclusion, our findings demonstrate that two markers, miR-487b and miR-516a-5p, enhance the accuracy of the current risk score, thus defining a better discrimination of neuroblastoma patients at diagnosis. Clinical use of this model may provide better therapeutic management of the disease by pediatric clinicians.

## Supporting Information

**Figure S1.** Kaplan–Meier curves comparing overall survival (respectively, disease-free survival) in the derivation and validation sets with the log-rank test.

**Figure S2.** Kaplan–Meier curves of expression of eight miRNAs of the 14q32.31 cluster—miR-380-5p, miR-494, miR-376c, miR-487b, miR-154, miR-134, miR-409-3p, and miR-410—for overall survival of the derivation set (*n* = 231). miRNAs expression levels were converted into discrete variables by discriminating the samples into two classes (high expression in blue and low expression in red), under or over the cutoff defined as the expression level of IGR-N-835 cell line taken as one.

**Figure S3.** Kaplan–Meier curves of expression of eight miRNAs of the 14q32.31 cluster—miR-380-5p, miR-494, miR-376c, miR-487b, miR-154, miR-134, miR-409-3p, and miR-410—for disease-free survival of the derivation set (*n* = 231). miRNAs expression levels were converted into discrete variables by discriminating the samples into two classes (high expression in blue and low expression in red), under or over the cutoff defined as the expression level of IGR-N-835 cell line taken as one.

**Figure S4.** Scatter-plots showing the statistical analysis of miR-516a-5p expression level relative to the expression level of IGR-N-835 cell line in the derivation set (*n* = 231) stratified in two groups, low-risk (blue, *n* = 151) versus high-risk (red, *n* = 80). Two-tailed Student's *t*-test indicates significance between the two subgroups. Black bars inside the plots indicate the mean value of miRNA expression levels.

**Figure S5.** Kaplan–Meier survival curves for overall survival and disease-free survival between four groups defined by the combination of miR-487b and miR-516a-5p in (A) low and (B) high standard risk groups of the derivation set (*n* = 231).

**Figure S6.** Area under the curve (AUC) of different prognostic models applied to the derivation set (*n* = 231).

**Figure S7.** Comparing observed (Kaplan–Meier) survival curves for risk groups (solid lines) and model-based predicted mean survival curves (dashed lines) (A) for overall survival and (B) for disease-free survival from the derivation set. Below each figure, the hazard ratio and 95% confidence interval into brackets of prognostic risk sets using group 1 as baseline were reported.

**Table S1.** Individual data of the 332 neuroblastoma patients (derivation and validation sets) and the expression levels of the two selected miRNAs using qRT-PCR.

**Table S2.** Cox proportional hazard models with backward selection of miRNAs from the C14MC cluster.

**Table S3.** Prognostic index (PI) for overall survival and disease-free survival estimated from the derivation set (*n* = 231) and cut-points used to define the risk groups in the validation set. The cut-points correspond to the median and third quartile of the distribution of the PI across patients from the derivation set.
