# Supplementary figures and images for: Expression of two parental imprinted miRNAs improves the risk stratification of neuroblastoma patients
Source: Cancer Med. 2014 Jun 13;3(4):998–1009. doi: 10.1002/cam4.264 (PMC4303168; doi:10.1002/cam4.264)

## Slide 1
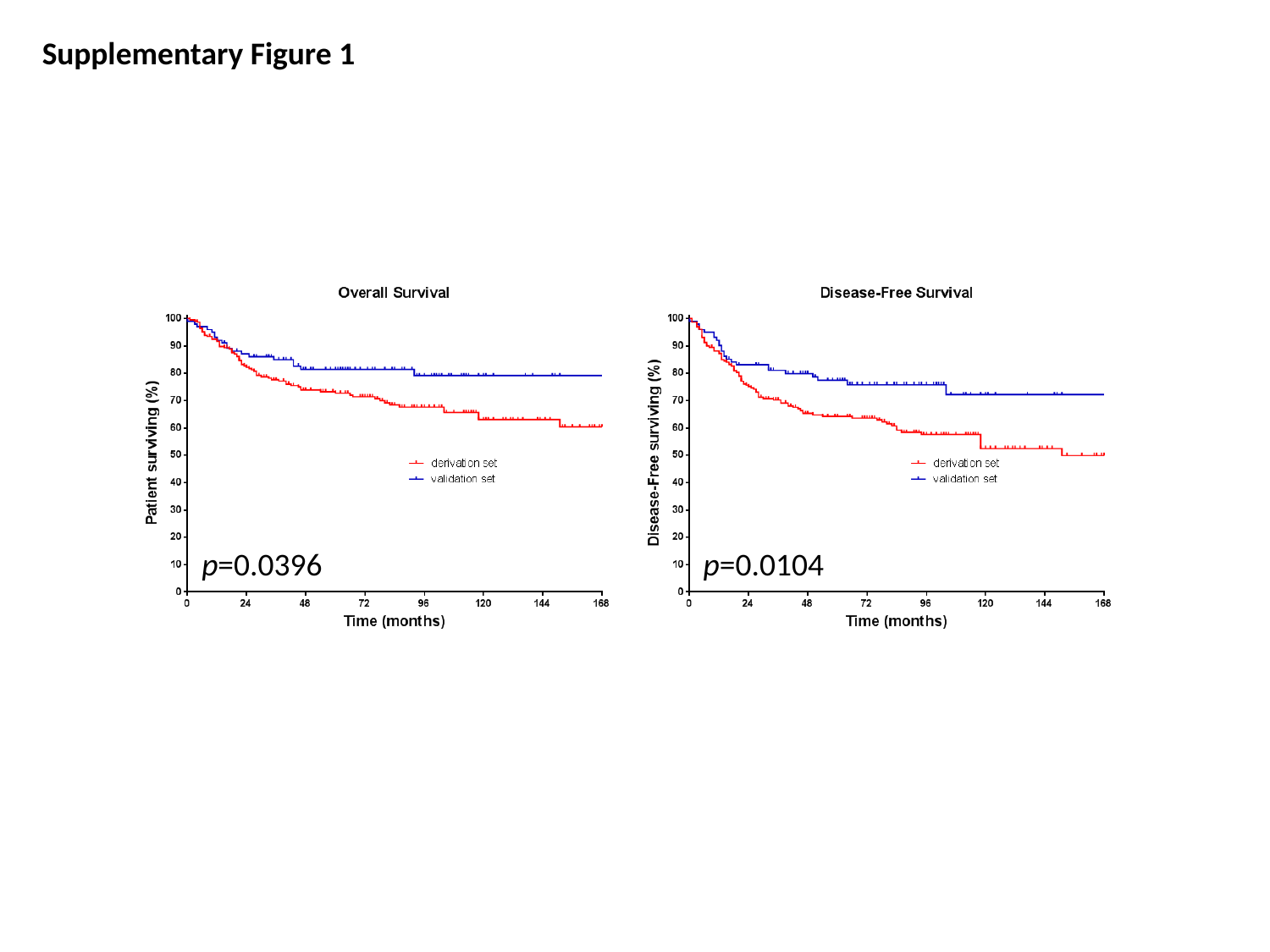

Supplementary Figure 1
p=0.0396
p=0.0104

Supplement: Supplementary file 1 [file cam40003-0998-sd1.pptx]

## Slide 1
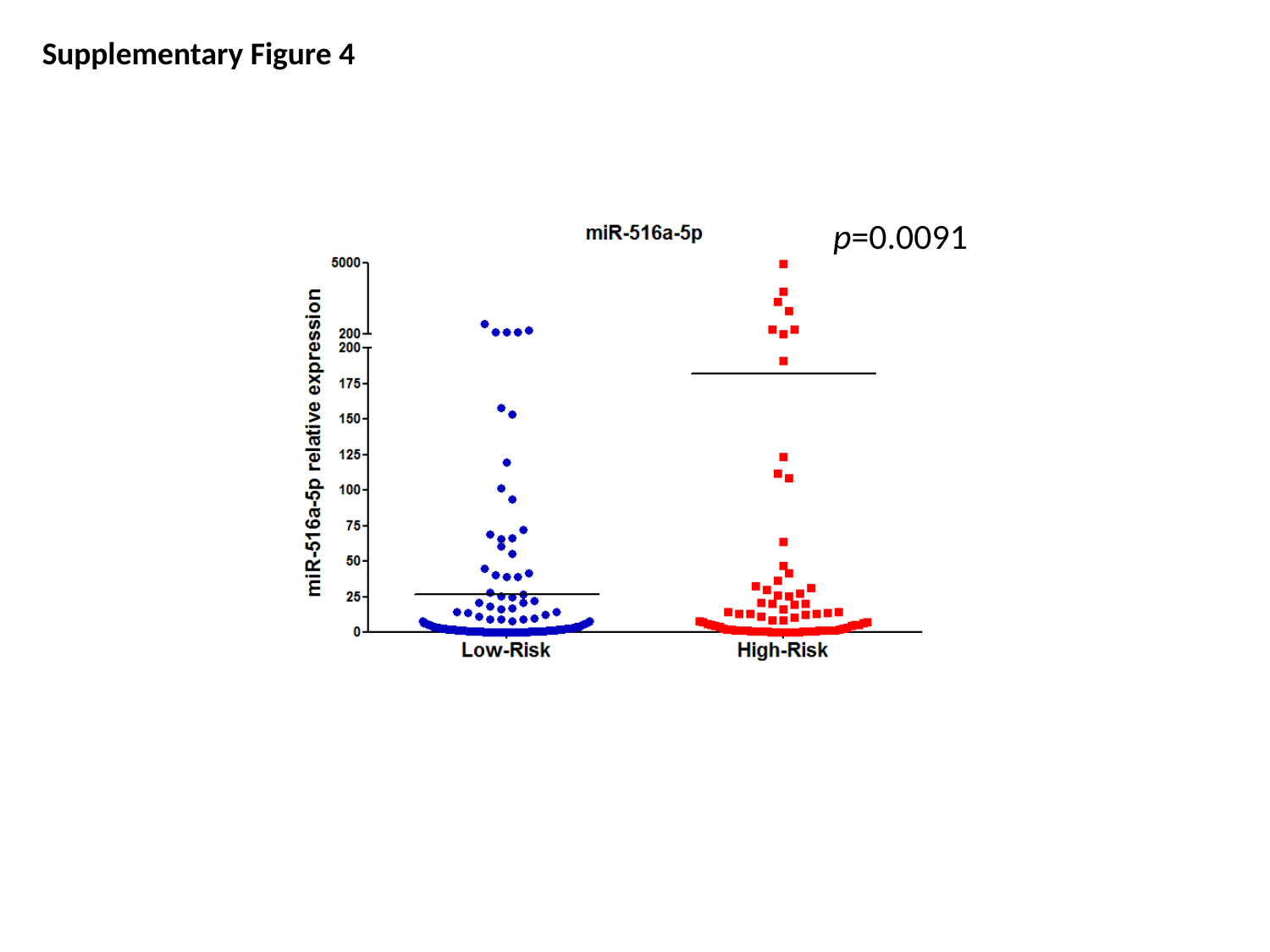

Supplementary Figure 4
p=0.0091

Supplement: Supplementary file 4 [file cam40003-0998-sd4.pptx]

## Slide 1
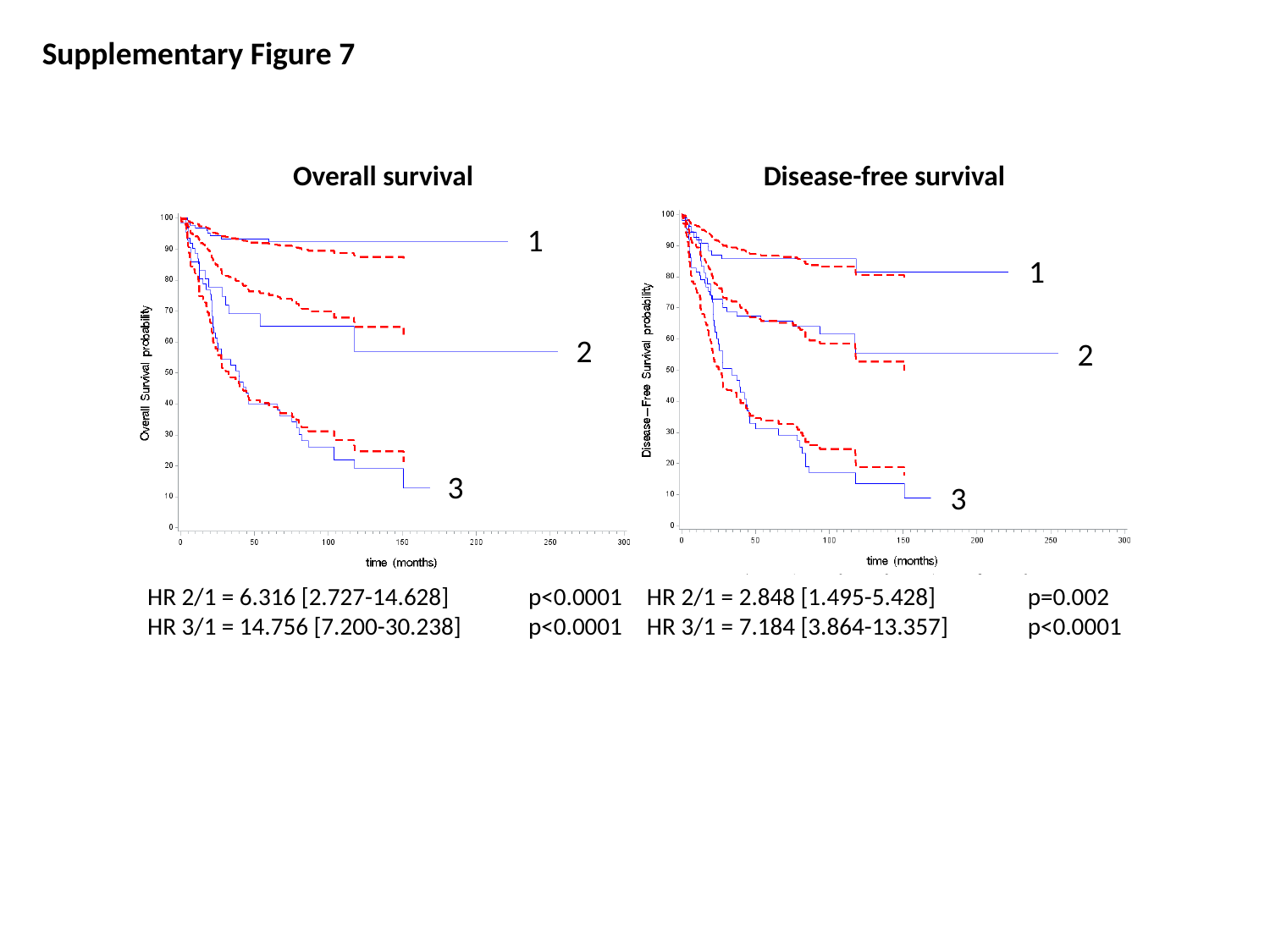

Supplementary Figure 7
Disease-free survival
Overall survival
1
1
2
2
3
3
HR 2/1 = 6.316 [2.727-14.628]	p<0.0001
HR 3/1 = 14.756 [7.200-30.238]	p<0.0001
HR 2/1 = 2.848 [1.495-5.428]	p=0.0020
HR 3/1 = 7.184 [3.864-13.357]	p<0.0001

Supplement: Supplementary file 7 [file cam40003-0998-sd7.pptx]
