# Supplementary material for: Expression of two parental imprinted miRNAs improves the risk stratification of neuroblastoma patients
Source: Cancer Med. 2014 Jun 13;3(4):998–1009. doi: 10.1002/cam4.264 (PMC4303168; doi:10.1002/cam4.264)
Supplement: Supplementary file 2 [file cam40003-0998-sd2.pptx]

## Slide 1
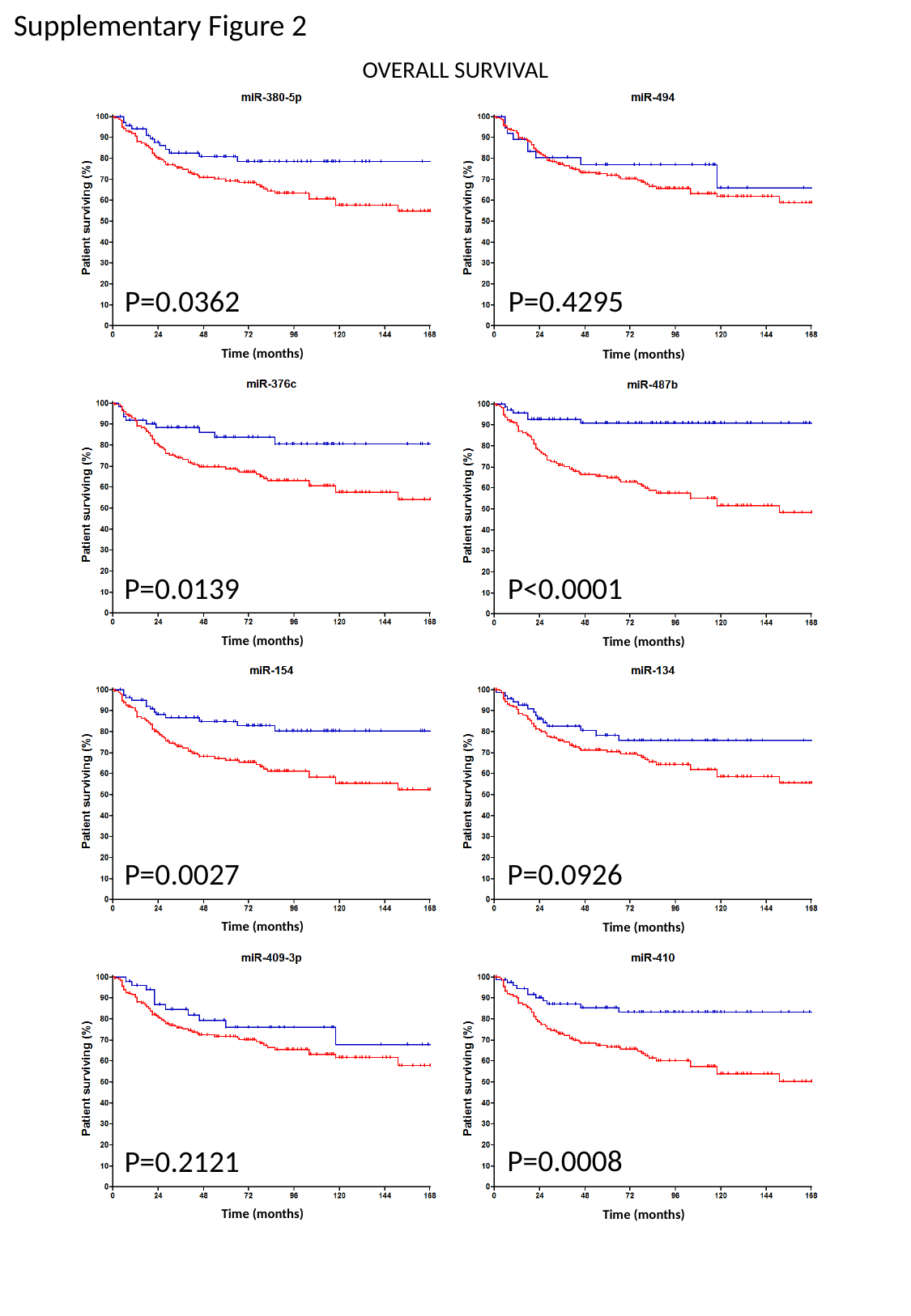

Supplementary Figure 2
OVERALL SURVIVAL
P=0.0362
P=0.4295
Time (months)
Time (months)
P<0.0001
P=0.0139
Time (months)
Time (months)
P=0.0027
P=0.0926
Time (months)
Time (months)
P=0.0008
P=0.2121
Time (months)
Time (months)
