# Supplementary material for: Expression of two parental imprinted miRNAs improves the risk stratification of neuroblastoma patients
Source: Cancer Med. 2014 Jun 13;3(4):998–1009. doi: 10.1002/cam4.264 (PMC4303168; doi:10.1002/cam4.264)
Supplement: Supplementary file 3 [file cam40003-0998-sd3.pptx]

## Slide 1
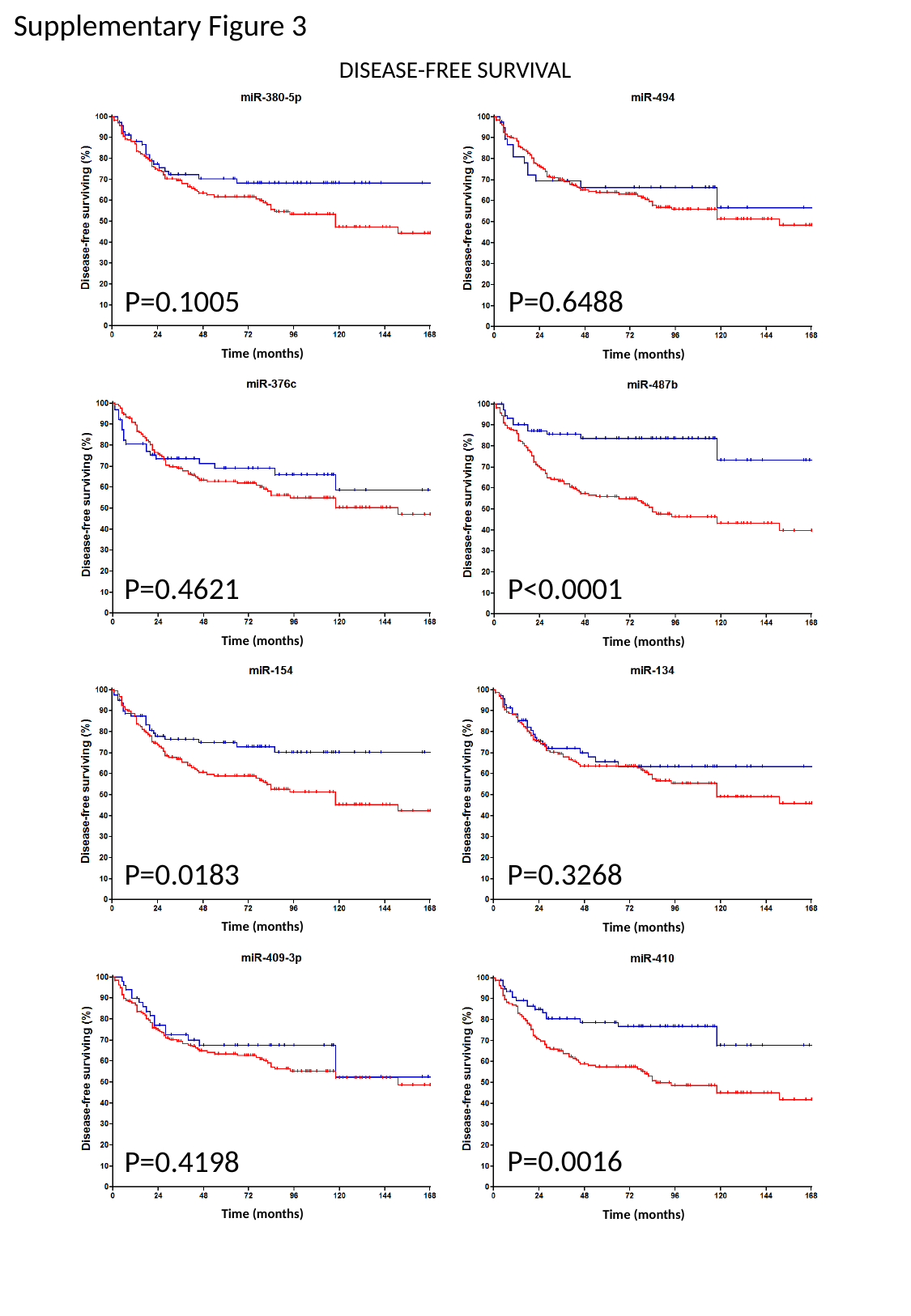

Supplementary Figure 3
DISEASE-FREE SURVIVAL
P=0.1005
P=0.6488
Time (months)
Time (months)
P<0.0001
P=0.4621
Time (months)
Time (months)
P=0.0183
P=0.3268
Time (months)
Time (months)
P=0.0016
P=0.4198
Time (months)
Time (months)
