# Supplementary material for: Expression of two parental imprinted miRNAs improves the risk stratification of neuroblastoma patients
Source: Cancer Med. 2014 Jun 13;3(4):998–1009. doi: 10.1002/cam4.264 (PMC4303168; doi:10.1002/cam4.264)
Supplement: Supplementary file 5 [file cam40003-0998-sd5.pptx]

## Slide 1
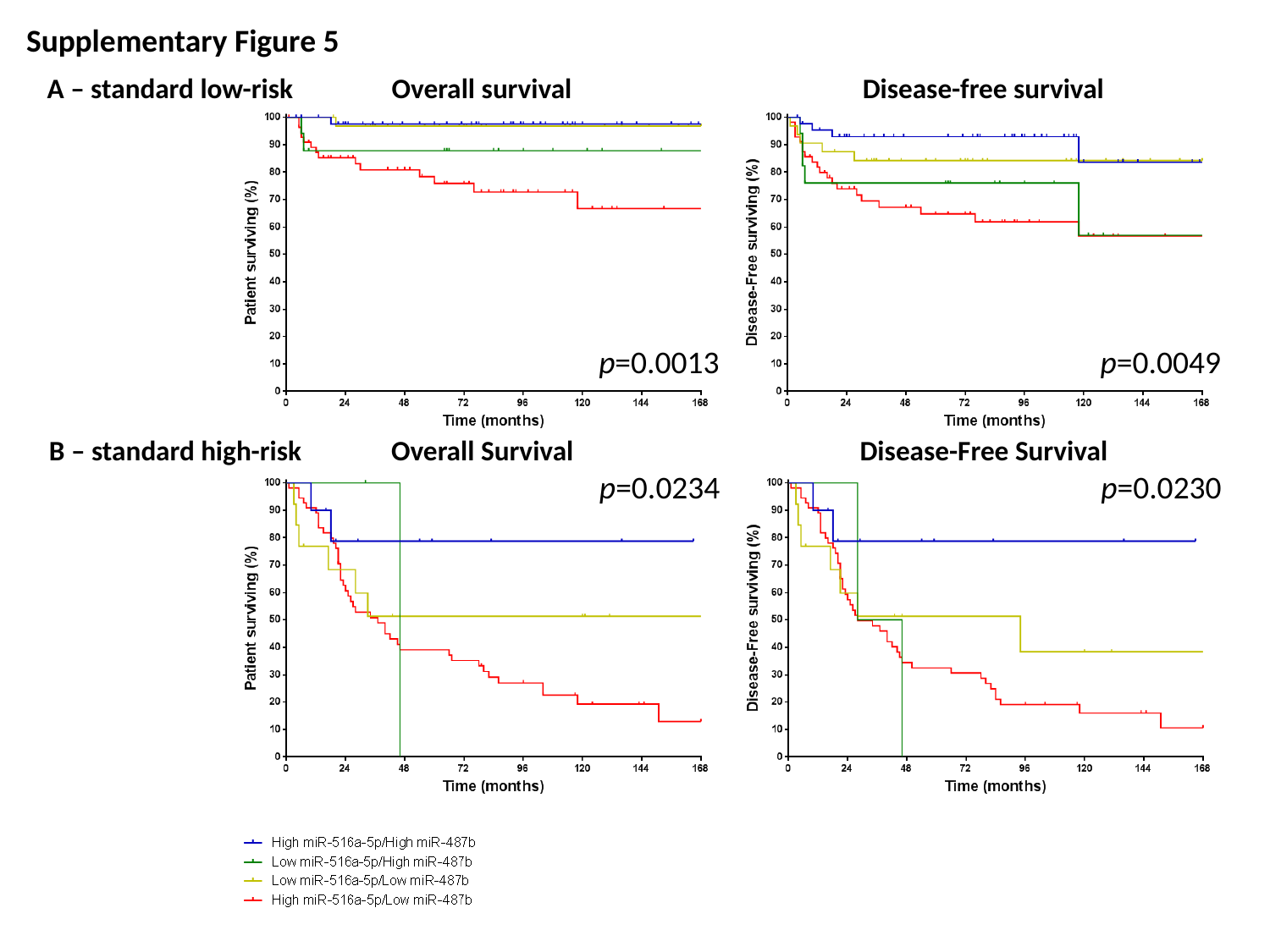

Supplementary Figure 5
A – standard low-risk
Disease-free survival
Overall survival
p=0.0013
p=0.0049
B – standard high-risk
Overall Survival
Disease-Free Survival
p=0.0234
p=0.0230
