# Supplementary material for: Expression of two parental imprinted miRNAs improves the risk stratification of neuroblastoma patients
Source: Cancer Med. 2014 Jun 13;3(4):998–1009. doi: 10.1002/cam4.264 (PMC4303168; doi:10.1002/cam4.264)
Supplement: Supplementary file 6 [file cam40003-0998-sd6.pptx]

## Slide 1
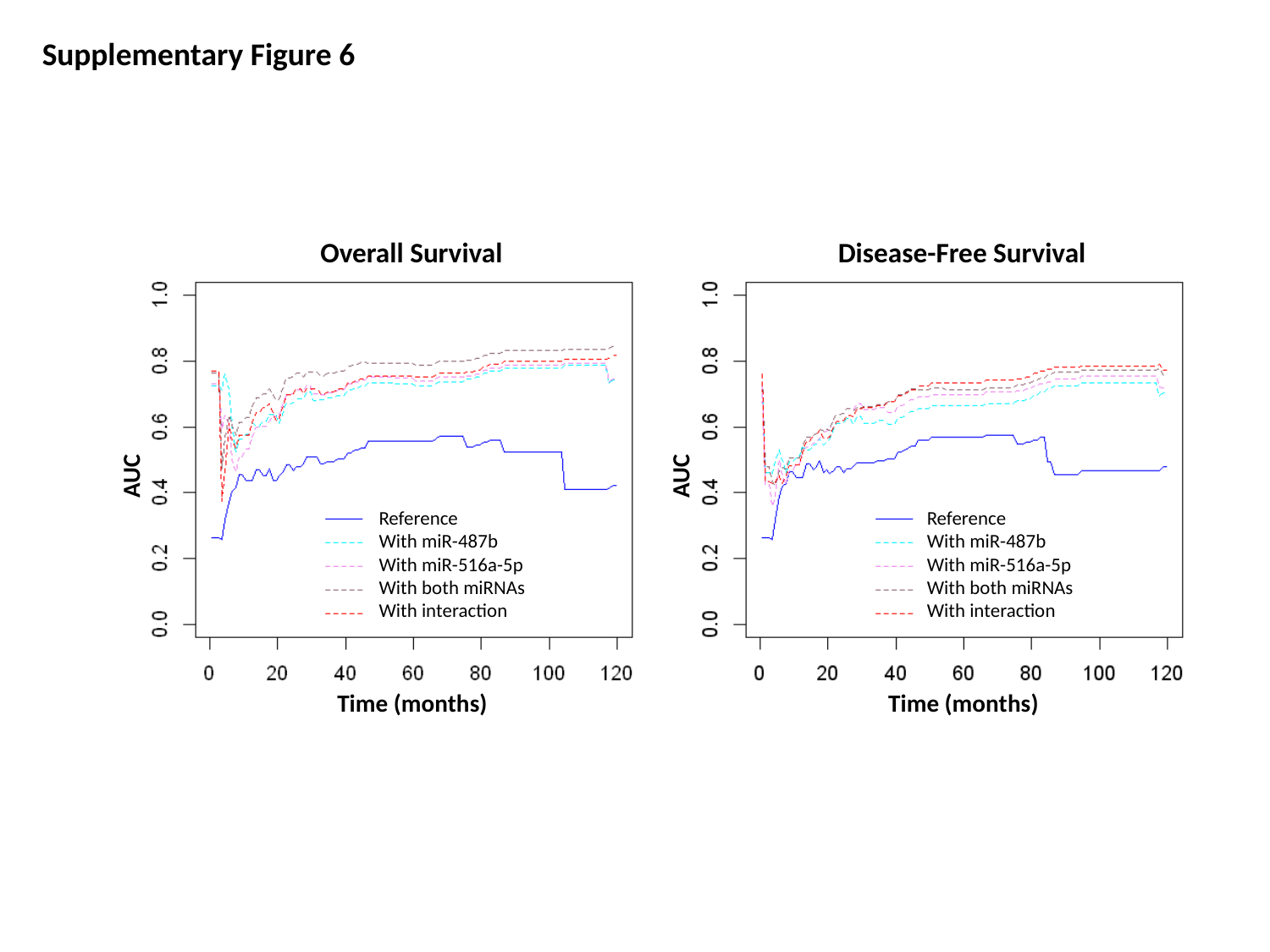

Supplementary Figure 6
Overall Survival
Disease-Free Survival
AUC
AUC
Reference
With miR-487b
With miR-516a-5p
With both miRNAs
With interaction
Reference
With miR-487b
With miR-516a-5p
With both miRNAs
With interaction
Time (months)
Time (months)
