# Supplementary material for: Expression of two parental imprinted miRNAs improves the risk stratification of neuroblastoma patients
Source: Cancer Med. 2014 Jun 13;3(4):998–1009. doi: 10.1002/cam4.264 (PMC4303168; doi:10.1002/cam4.264)
Supplement: Supplementary file 9 [file cam40003-0998-sd9.docx]

**Supplementary Table 2**.

Cox proportional hazard models with backward selection of miRNAs from the C14MC cluster

| **Characteristics** | **Overall Survival** | | **Disease-Free Survival** | |
| --- | --- | --- | --- | --- |
|  | **Hazard Ratio [95%CI]** | ***p*-value** | **Hazard Ratio [95%CI]** | ***p*-value** |
| Age | 1.849 [0.980-3.491] | 0.0579 | 1.511 [0.902-2.530] | 0.1165 |
| Stade | 4.013 [2.127-7.570] | <0.0001 | 2.657 [1.586-4.452] | 0.0002 |
| *MYCN* amplification | 1.581 [0.907-2.756] | 0.1058 | 1.265 [0.744-2.150] | 0.3861 |
| miR-380-5p |  | 0.5867 |  | 0.1332 |
| miR-494 | 2.670 [1.178-6.054] | 0.0187 |  | 0.7825 |
| miR-376c |  | 0.3991 | 2.635 [1.452-4.782] | 0.0014 |
| miR-487b | 0.219 [0.084-0.568] | 0.0018 | 0.278 [0.138-0.559] | 0.0003 |
| miR-154 |  | 0.3331 |  | 0.3761 |
| miR-134 |  | 0.7406 |  | 0.5863 |
| miR-409-3p |  | 0.6194 |  | 0.3602 |
| miR-410 |  | 0.5408 |  | 0.9730 |

These results, however, may be wobbly due to the correlation between miRNAs. We thus performed a robustness analysis based on the replication of 1000 bootstrapped samples from the 231 patients. Each bootstrapped-sample was analysed using a multivariable Cox model and we counted the number of time that each miRNA was significantly associated with OS and DFS. miR-487b was the miRNA the most often selected (data not shown).
