# Supplementary material for: Expression of two parental imprinted miRNAs improves the risk stratification of neuroblastoma patients
Source: Cancer Med. 2014 Jun 13;3(4):998–1009. doi: 10.1002/cam4.264 (PMC4303168; doi:10.1002/cam4.264)
Supplement: Supplementary file 10 [file cam40003-0998-sd10.docx]

**Supplementary Table 3.**

Prognostic index (PI) for OS and DFS estimated from the derivation set (n=231) and cutpoints used to define the risk-groups in the validation set. The cutpoints correspond to the median and third quartile of the distribution of the PI across patients from the derivation set.

| Outcomes | Prognostic index | Cutpoints |
| --- | --- | --- |
| OS | 0.671*age + 1.107*stage + 0.473*mycn + 0.604*miR_487b_in miR-516a-5p low -1.791* mir_487b_ in miR-516a-5p high + 1.020*miR-516a-5p | Group 1: PI<1.020065 |
|  |  | Group 2: 1.020065 ≤ PI< 2.127662 |
|  |  | Group 3: PI ≥ 2.127662 |
| DFS | 0.442*age + 0.738*stage + 0.249*mycn + 0.741*miR_487b_in miR-516a-5p low -1.517* mir_487b_ in miR-516a-5p high + 0.715*miR-516a-5p | Group 1: PI<0.715645 |
|  |  | Group 2: 0.715645≤ PI< 1.454237 |
|  |  | Group 3: PI ≥ 1.454237 |
